# Hepatitis B vaccination coverage across India: exploring the spatial heterogeneity and contextual determinants

**DOI:** 10.1186/s12889-019-7534-2

**Published:** 2019-09-12

**Authors:** Junaid Khan, Apurba Shil, Sanjay K. Mohanty

**Affiliations:** 1 International Institute for Population Sciences, Govandi Station Road, Deonar, Mumbai, 400088 India; 2 Department of Public Health, Faculty of Health Sciences, Ben Gurion University of the Negev, Be’er Sheva, Israel; 3 Department of Fertility Studies, International Institute for Population Sciences, Govandi Station Road, Deonar, Mumbai, 400088 India

## Abstract

**Background:**

Although hepatitis B vaccinations have been integrated in the Universal Immunization Program (UIP) in India over a decade, only half of the children are immunized against hepatitis B. The national average in hepatitis B vaccination conceals large variations across states, districts and socio-economic groups. In this context, the aim of this paper is to examine the spatial heterogeneity and contextual determinants of hepatitis B vaccination across the districts of India.

**Methods:**

Using data of 199,899 children aged 12–59 months from the National Family Health Survey-4 (NFHS-4), 2015–16 we have examined the district level spatial distribution and clustering of hepatitis B vaccination with the help of Moran’s I and Local Indicator of Spatial Autocorrelation (LISA) measures. We investigated the low coverage of HBV vaccination using spatial autoregressive models (SAR) at the meso scale. And we applied multivariate binary logistic regression analysis to understand the micro-level predictors of hepatitis B vaccination.

**Results:**

In 2015–16, 45% of the children aged 12–59 months were not vaccinated against hepatitis B in India. The coverage of hepatitis B vaccine across the districts of India showed a highly significant spatial dependence (Moran’s *I* = 0.580). Bivariate Moran’s *I* confirmed the spatial clustering of hepatitis B vaccination with mother’s education, full antenatal care (ANC) utilization, post natal care (PNC) utilization, institutional births and registration of births at the district level. Districts with a very low coverage of HBV vaccine are clustered in the western, north-eastern regions and in some parts of central India. At the unit (child) level, children’s hepatitis B immunization status is mostly determined by the socio-economic and demographic characteristics like their mother’s educational status, caste, religion, household’s wealth condition, birth order, year of birth and the region they belong to.

**Conclusions:**

District level variation in hepatitis B vaccination is spatially heterogeneous and clustered in India with a strong neighbourhood effect. Uptake of hepatitis B vaccine among Indian children is predominantly dependent upon their socio-economic and demographic characteristics.

## Background

Hepatitis B virus (HBV) infection is a major public health challenge in developing countries. The morbidity and mortality pattern due to HBV infection is close to the severity of HIV/AIDS endemicity [1, 2]. Although immunization remains the most effective way to control the spread of HBV infection, it is estimated that every year at least 27 million children worldwide do not receive the basic doses of immunizations [3]. According to World Health Organization (WHO), one-third of the global population (two billion people) have been infected with hepatitis B virus. In 2013, other viral hepatitis accounted for 1.45 million deaths with 63% increased burden of deaths than that from 1990 of 0.89 million deaths [4]. The prevalence of hepatitis B virus varies between 5 to 20% in the developing countries [5].

Vaccination against hepatitis through safe injection during early childhood is very important to prevent the infection among children and during their life course. In 1991, the Expanded Program on Immunization (EPI) by WHO recommended to introduce hepatitis B vaccination in the routine infant immunization to prevent the infection during early childhood [2]. Identifying the severity of hepatitis infection, about 175 WHO member countries integrated hepatitis B vaccination in their national immunization program by the year 2009 [6].

India carries the second largest burden of chronic HBV infections globally [5–7]. About 50 million people are chronic HBV carriers in India and the prevalence of hepatitis B surface antigen (HBsAg)^1^ ranges between 2 to 8% in general population [5, 8, 9]. India belongs to “intermediate to high endemicity” group of countries for hepatitis B surface antigen constituting approximately 11% of the estimated global burden [2, 10]. Every year, around 115, 000 Indians die due to HBV infections and complications [11]. Due to poor hygiene and population density in India, children are more susceptible to the infection. Around 1 million children (out of 26 million) born every year are at risk of developing chronic HBV infection during their life time [12]. It is found that vertical transmission is very negligible and horizontal transmission largely contributes to HBV infection in India [13].

In India, hepatitis B vaccination was launched in the year 2002 mainly in urban India (14 metropolitan cities) and in 2003, it extended to 33 additional rural districts [10]. Finally the Indian National Policy (UIP) on immunization recommended vaccinating the children with three doses of hepatitis B along with the other six vaccine preventable diseases (VPDs)-Polio, Diptheria, Pertusis, Tetanus, Tuberculosis and Measles. Universal immunization program (UIP) in India is one of the centrally sponsored programmes that aim to immunize every child in the country against VPDs [14]. The current immunization schedule in India includes a birth dose within 24 hours for all the institutional deliveries to prevent the perinatal transmission. But irrespective of the birth dose, three doses are given to the new born at 6, 10 and 14 weeks along with oral polio virus (OPV) and DPT for a complete immunization against hepatitis B taking care of the large number of non-institutional births [15].

There are small scale and unrepresentative studies that examined the variations and determinants of hepatitis B vaccination in India [16, 17]. To our knowledge, there is no population based study that examined the spatial pattern and contextual determinants of hepatitis B vaccination in India, possibly due to data constraints [2, 18]. The National Family Health Survey (NFHS)-4, 2015–16, fourth in the series of Demographic Health Surveys (DHS) for India for the first time collected the information on hepatitis B vaccine in the country. Using the data of NFHS-4, this study assessed the spatial pattern and contextual determinants in the coverage (percentage of children received the three doses of hepatitis B) of hepatitis B vaccine among children aged 12–59 months across the districts of India.

## Methods

### Data source and sampling

The study is a cross sectional study and utilized the data from NFHS-4 which is publicly available through (https://dhsprogram.com/data/dataset/India_Standard-DHS_2015.cfm?flag=1). Thus, no further ethical approval is required. NFHS 4 was conducted during January 20, 2015 and December 4, 2016 across 640 districts spread over 36 states and union territories of India. Districts in India are the second basic and policy relevant administrative units. NFHS 4 is the Indian version of Demographic Health Survey (DHS) that used standard survey instruments across the country. NFHS used a stratified two stage cluster design to conduct the survey and used the 2011 census sampling frame to select the primary sampling units (PSU). Census enumeration blocks in urban areas and villages in rural areas constitute the sampling frame of PSUs. PSUs were then selected using probability proportional to size (PPS) from the sampling frame. Prior to the main survey, a complete household mapping and listing was done in the selected rural and urban PSUs and within the selected PSUs, the number of households (300 at least) were sub divided into segments of 100–150 households. And finally two of the segments were randomly selected using systematic sampling with probability proportional to segment size. In the second stage, 22 households were randomly selected with systematic sampling from the rural and urban clusters of segments. The details of sampling design, instrument and survey findings are available for public use [19]. The study sample consists of 199,889 children aged 12–59 months. Of the total 211,773 children, information on the three doses of HBV vaccine was not available for 11,884 children and these observations were dropped from the study.

### Identification of the children with hepatitis B vaccination uptake

During the survey, as a part of the core questionnaire, the mothers were asked to show the vaccination card to collect the information on various doses of vaccination including hepatitis B. In case mothers could not show or did not have vaccination card at the time of survey, they were asked whether the child received the doses of hepatitis B vaccine. A child is said to be vaccinated against hepatitis B if he/she was found vaccinated either in card or from mothers reporting. Those mothers who reported “Do not Know” were treated as not vaccinated (1.8% of total cases). This is the standard recommendation by Demographic Health Survey (DHS) to estimate the vaccination coverage among the children [20]. Among 199,889 children aged 12–59 months, only 109, 085 received all the three doses of hepatitis B vaccine. Although NFHS collects the information on the birth dose of hepatitis B, but NFHS provides an estimate of children receiving the three doses of hepatitis B received at 6, 10 and at 14 weeks from the day of birth, independent of the birth dose. As the present study is based upon NFHS data, we considered the last three doses of hepatitis B being received at 6, 10 and 14 weeks to create the outcome variable in this study. Table 6 in Appendix provides hepatitis B vaccine uptake information for all the three doses other than the birth dose among the study children.

It is likely to be some recall bias (non-sampling bias) in the data and it could be in either direction-over reporting or under reporting. Although, checking the validity of mother’s recall was beyond the scope of the study but to reduce the non sampling bias due to mother’s recall, we controlled the socio-economic and demographic factors which mostly determine the pattern of recall bias among mothers [21]. In another account to take care of the sampling bias, we used the “svy” command in Stata version 12.0 SE (STATA Corp LP, College Station,TX) with sampling weights to address the corresponding sampling bias and to get the unbiased estimates.

The analyses have been carried out at district level and at individual level (child). Prior to unit level analyses, the district level analysis is a comprehensive effort to understand the analogy of district level coverage of hepatitis B vaccination and its determinants in a spatial setting because, after states, district is the second administrative and policy relevant unit where demographic events and population health indicators are estimated to track and monitor the health conditions of the general population in India.

### Outcome variable

The outcome variable for the district level analysis is the proportion of children aged 12–59 months who received 3 doses of hepatitis B vaccine. In case of child level analyses, the outcome variable is the hepatitis B vaccination status of the child (whether immunized against hepatitis B or not). A child who was given all the three doses of hepatitis B vaccines considered to be vaccinated against hepatitis B virus otherwise not. Thus, the hepatitis B vaccination status of a particular child is a binary variable where ‘1’ is yes which denotes the child received all the three doses and ‘0’ otherwise.

### Independent variables

A set of socio-economic and demographic indicators at the district level were used to predict the coverage of hepatitis B vaccine. These include the - (1) percentage of women with 10 or more years of schooling, (2) percentage of mothers who had full antenatal care (ANC), (3) percentage of mothers who received postnatal care (PNC) from a doctor/nurse/LHV/ANM/midwife/other health personal within 2 days of delivery (4) percentage of mothers who received financial assistance under Janani Suraksha Yojana (JSY) scheme for births delivered in an Institution, (5) percentage of institutional births (6) percentage of households with electricity connection, (7) percentage of households with an improved drinking-water source,^2^ (8) percentage of children under age 5 years whose birth was registered and (9) percentage of breastfeeding children receiving an adequate diet.^3^

### Spatial analyses

District level variations and determinants were examined using spatial analyses. According to Census of India 2011, there are 640 districts across 36 states and union territories with an average population size of 2 million [22]. These districts vary enormously in demographic, social, economic and health indicators. NFHS-4 for the first time had the distinction of providing demographic and health estimates at the district level.

To understand the spatial clustering of immunization across districts, Local Moran’s *I* indices were generated to measure the spatial autocorrelation. Similarly, bivariate LISA was used to analyze the association of certain characteristics of regions (districts) with the hepatitis B vaccine coverage across those districts. Such analyses has been increasingly used to understand the spatial heterogeneity in terms of demographic and public health indicators across the population [23]. Moreover, district level spatial analyses are helpful to assess the geographical disparity in health or other concerned indicators and identify the geographical pockets underprivileged in terms of the same [23, 24]. To check the empirical associations between the outcome and independent variables of the study, we preliminarily estimated the ordinary least square (OLS) model and conducted spatial diagnostics of the residuals in OLS model. As the event of study showed a statistically significant Moran’s I, we built up the spatial autoregressive models-spatial lag and spatial error model.

District level quintile maps were generated using Arc-GIS to understand the spatial pattern of child immunization coverage in India. Queen’s contiguity method of order 1 was used to create the spatial weight matrix (*w*) in the analyses. Arc-GIS version 10.1 and Geo-Da version 1.8.16.4 were used for the spatial analyses.

### India digital map

The India shape file was obtained from GitHub through https://github.com/datameet/maps/tree/master/Districts and was used under the Creative Commons Attributions 2.5 India license. The projection of the map was in WGS 1984 UTM zone 43 N.

### Unit (child) level analyses

The set of independent variables used in the unit level analyses are child level characteristics, maternal characteristics and household characteristics. The child level characteristics include age of the child (in months), year of birth, sex, birth order, child lives with whom. Age of the child is categorized into four groups (12–23, 24–35, 36–47 & 48–59) while the year of births of the study children are 2010, 2011, 2012, 2013, 2014 & 2015. The birth order refers to the order of the child among all live births to a mother and labeled as first, second, third, fourth etc. Previous studies also explored the variation in child health care utilizations in terms of the birth order of the child [25]. Sex of the child is another important variable considered in this study to find the gender differential of hepatitis B vaccine coverage among the study group of children. Sex is a bio-demographic characteristic of the child and children are classified as male and female. To understand the care given to the children and health care utilization for the children it is important to know whether the child lives with their mother or not. Although we did not find any previous study but we assume that children living with a parent are likely to receive better care. Here the variable is categorized into two following categories- children living with mother & lives elsewhere. The maternal characteristics included in the analyses are mother’s educational attainment, caste and religion. Mother’s educational attainment is classified as no education, up to primary educated, completed secondary education and higher secondary or more educated. Caste is another important social variable that depicts the economic and social well being of the households in India. In India, the population is classified into four caste groups, namely, scheduled caste (SC), scheduled tribe (ST), other backward class (OBC) and others. Among these groups, SC & STs are the most under privileged and secluded groups in India. The national, state and local government in India provides reservation benefits to SC, ST and OBCs in education, employment, health and other related programs. Similarly, we have used four religion groups (Hindu, Muslim, Christianity and others) in the analyses and these three are the major religious groups in India. Wealth quintile in DHS data is a measure to capture the economic well being of the household. It is derived from a set of 37 asset based variables using principal component analysis (PCA). For analytical purpose, the wealth index is grouped into five categories-poorest, poor, middle, richer & richest. Besides, we have used place of residence (rural/urban) and region (North, South, Central, West, East and North East) in the analyses. These classifications are similar to that of NFHS-4 [26].

### Bivariate and multivariate regression analyses

Bivariate analysis was used to understand the differentials in vaccination coverage by socio-demographic characteristics. The conditional probabilities were estimated for hepatitis B vaccination conditioned on the background characteristics of the children. And the multivariate logistic regression was used to understand the determinants of hepatitis B vaccination at individual level. Child’s hepatitis B immunization status (received all the three doses of the vaccine -yes/no) has been modeled and adjusted to a set of independent factors. A total of 1, 99,889 children aged 12–59 months consisted the unit level analysis of this study. Stata version 12.0 SE (STATA Corp LP, College Station,TX) was used to analyse the data.

## Results

### District level

**Fig. 1:**
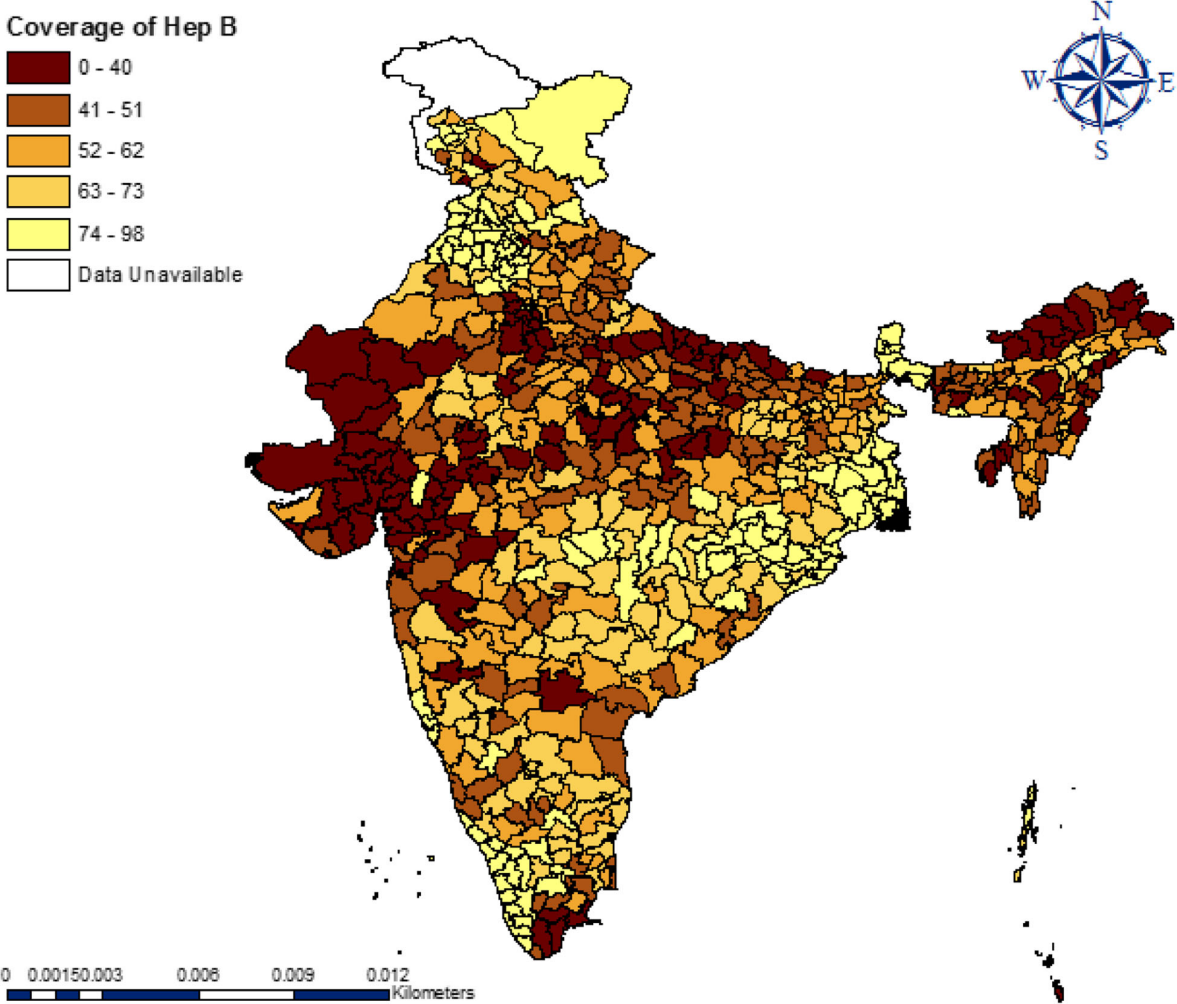
Quintile maps showing the spatial distribution of hepatitis B vaccination coverage across districts of India, 2015–16 (NFHS-4)

Figure 1 gives the quintile map showing the distribution of hepatitis B vaccination across districts of India. A total of 121 districts had hepatitis B vaccination coverage of less than 41% (dark brown), 135 districts had between 41 and 51%, 154 districts between 52 and 62% and a total of 230 districts had more than 62% coverage. Geographical disparities and the gradual spatial progression from low to high can be observed in the coverage of hepatitis B across the districts. A high coverage of hepatitis B vaccine is highly clustered in southern and south-eastern parts of India. Very low coverage of hepatitis B vaccine can be observed mainly in western, north-eastern regions and some parts of central India. Table 7 in Appendix provides the spatial dependence of hepatitis B vaccine coverage and each of the indicators across the districts of India. And the corresponding Moran’s *I* value indicates the neighborhood effect.

**Fig. 2:**
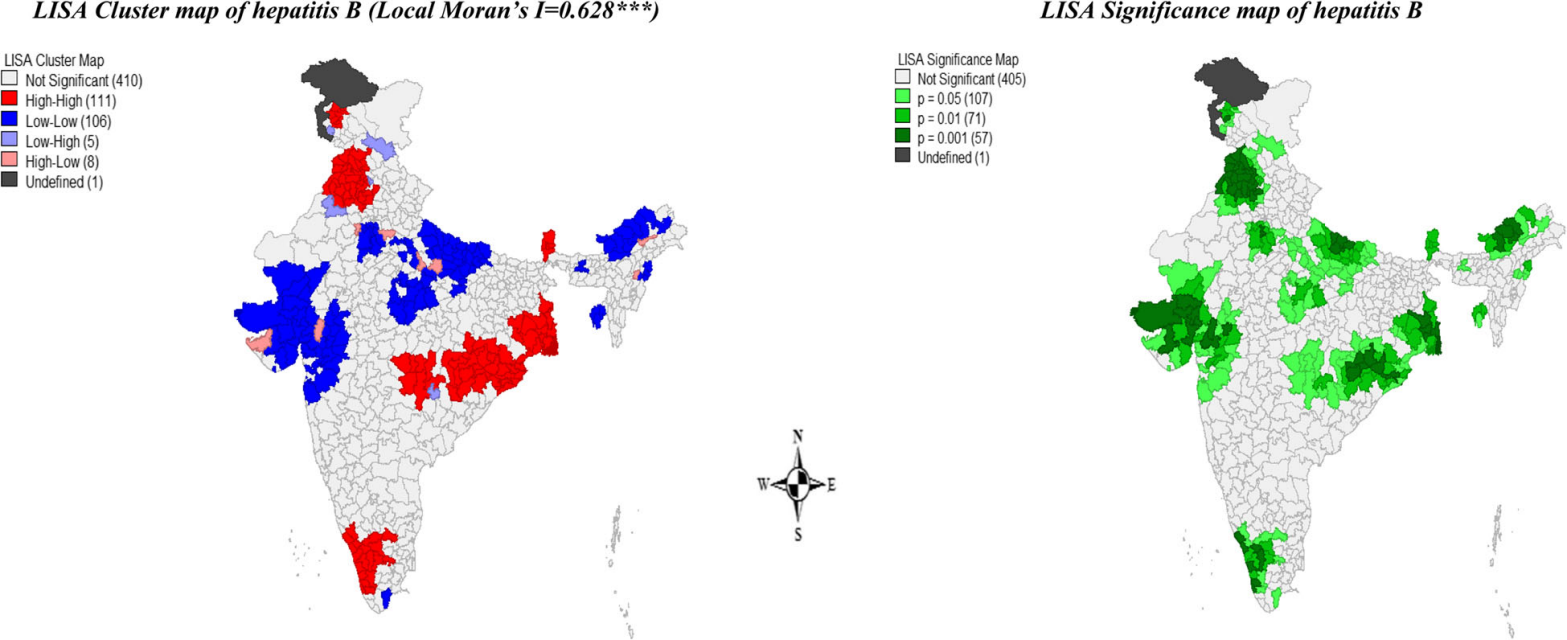
Univariate LISA Cluster maps showing the spatial clusters and spatial outliers of hepatitis B vaccination coverage across districts of India, 2015–16 (NFHS-4)

Figure 2 presents univariate LISA cluster and significance maps of hepatitis B vaccine coverage in districts of India. The Moran’s *I* statistic was 0.628, suggested a highly significant spatial dependence in the coverage of hepatitis B vaccination in India. A total of 106 districts from Gujarat, Rajasthan, some parts of Uttar Pradesh and North-Eastern states formed the cold spots (low-low coverage) while 111 districts from West Bengal, Punjab, Himachal Pradesh, Orissa, some parts of Haryana, Chhattisgarh, Andhra Pradesh, Maharashtra, Kerala, Tamil Nadu and Telangana formed the hot spots (high-high coverage) across India. A total of 13 districts have been found as spatial outliers (high-low or low-high) of hepatitis B vaccination coverage in the country.

Table 1 presents the bivariate spatial association of hepatitis B vaccine with the socio-economic and demographic indicators. District level percentage of full ANC, PNC, women with 10+ years of schooling, institutional delivery and birth registration showed a dominant and statistically significant association with the coverage of hepatitis B vaccination among the children across the districts of India. The value of Moran’s *I* was lower for ‘mother’s receiving financial assistance’, ‘safe drinking water’ and ‘breastfed child receiving adequate diet’. This indicated that utilization of maternal health care utilization among the mothers across districts is substantially associated with HBV vaccination coverage among the children.

**Table 1:** Moran’s *I* statistics of Hepatitis B vaccine coverage and socio-economic indicators in districts of India, 2015–16

Bivariate Local Moran’s I statistic with significance level	
Indicators	Hepatitis B vaccine
Women with 10+ years of schooling (%)	0.30 (0.00)
Full ANC (%)	0.36 (0.00)
PNC (%)	0.40 (0.00)
Mothers who received financial assistance (%)	0.03 (0.03)
Institutional Births (%)	0.28 (0.00)
Electricity (%)	0.24 (0.00)
Drinking water (%)	0.07 (0.00)
Births Registered (%)	0.30 (0.00)
Breastfed child receive adequate diet (%)	0.17 (0.00)

Table 2 gives the estimated results from the ordinary least square (OLS), SLM and SEM model for hepatitis B vaccine in the districts of India. OLS model gives the adjusted estimates without considering the spatial correlation into account whereas the spatial auto regressive (SAR) models give the association between the predictors and hepatitis B vaccine coverage by considering the spatial effects into account. In all the three models, we found education of mother, utilization of full ANC, PNC and financial assistance to be the highly statistically significant predictor of HBV vaccine coverage across the districts of India. Based on the model diagnostics of the spatial models, SLM model gave the best fit to the data under study with the lowest AIC value. The SLM model showed that except ‘birth registration’, ‘drinking water’ and ‘breastfed child receiving adequate diet’ all the predictors have statistically significant association with hepatitis B vaccination coverage. In the SLM model, the coefficient of PNC (β = 0.33, *p*-value< 0.01) was the largest followed by full ANC (β = 0.15, *p*-value< 0.01), mothers received financial assistance (β = 0.12, *p*-value< 0.01) and education of the women (β = 0.12, *p*-value< 0.01). This indicated that post natal care utilization among mothers across districts is strongly associated with the rate of immunization (hepatitis B) among the children in India. Interestingly, in the first model (OLS model) proportion of breastfed child receiving adequate diet showed a significant association with the coverage of HBV vaccine but once the spatial autocorrelation was adjusted the effect became insignificant in the SLM as well as in the SEM model.

**Table 2:** Results of OLS, Spatial Lag model (SLM) & Spatial Error Model (SEM) estimation of hepatitis B vaccination in districts of India, 2015–16

Predictors	Hepatitis B Vaccination*		
	OLS	SLM	SEM
Women schooling 10+ years (%)	0.24 (0.00)	0.12 (0.00)	0.16 (0.01)
Full ANC (%)	0.25 (0.00)	0.15 (0.00)	0.25 (0.00)
PNC (%)	0.57 (0.00)	0.33 (0.00)	0.27 (0.00)
Mothers received financial assistance (%)	0.20 (0.00)	0.12 (0.00)	0.15 (0.00)
Institutional Births (%)	−0.26 (0.00)	−0.11 (0.00)	−0.01 (0.79)
Electricity (%)	−0.09 (0.0.06)	−0.09 (0.00)	− 0.14 (0.01)
Drinking water (%)	−.02 (0.72)	−0.01 (0.65)	− 0.10 (0.02)
Births Registered (%)	0.01 (0.13)	0.02 (0.59)	0.01 (0.81)
Breastfed child received adequate diet (%)	0.11 (0.04)	0.09 (0.12)	0.11 (0.13)
N	640	640	640
ρ		0.62 (0.00)	
λ			0.71 (0.00)
AIC	5085.6	4871	4893
Adjusted R^2^	0.450	0.67	0.67

* Each cell shows the corresponding regression coefficient and the *p*-value within parentheses

### Individual level (child level)

After a- district level meso scale examination of the hepatitis B vaccine coverage in the previous section, this section describes the coverage of hepatitis B vaccination at individual level and it’s socio-economic and demographic determinants among the children across India.

In the study sample we found that 48% of the children were female and 52% were male. Of the total children, 76% of the children were from rural areas whereas 24% were from urban areas. A significant portion (26.5%) of the children belongs to the poorest wealth quintile. Around 72% of children were Hindu, 8% were Christian and 16% were Muslim. About 20% belonged to the scheduled caste and 21% belonged to the scheduled tribe (ST) group. The study population of the children were found to be of different birth orders. Around 37% of the children were of the first birth order whereas 17% of them were found to be of fourth or higher orders. And the mean age of these children was 35.5 months.

Table 3 gives the coverage of HBV vaccine in India, Gujarat and Punjab by background characteristics. Punjab had the highest and Gujarat had the lowest coverage of hepatitis B vaccine. The vaccination coverage did not show large variation across rural and urban areas. The variation in hepatitis B vaccination was large by wealth quintile. Children from the poorest section showed the lowest coverage than the rest of the wealth groups with a distinct gradient of higher vaccination rate over the improved wealth status categories. For example, the coverage of hepatitis-B vaccine was 47% in poorest wealth quintile compared to 64% in richest wealth quintile and the pattern was similar in Gujarat and Punjab. Religion differential of HBV vaccine was also evident. In the state of Gujarat, we found that the coverage was highest among Christian children (58%) compared to Hindu and Muslim children. Overall, this state showed a comparatively lower vaccination rate among the different religious group of children than the national rate. It was observed that Muslim children carried the burden of lowest coverage of HBV vaccination among them.

**Table 3:** Hepatitis B vaccination coverage by socio-demographic characteristics; India, Gujarat^d^ and Punjab^e^, NFHS, 2015–16

Background characteristics	India		Gujarat		Punjab	
	Hep-B (%)	No. of Children	Hep-B (%)	No. of Children	Hep-B (%)	No. of Children
Child’s age in months						
12–23	63	48,928	39	1397	91	1018
24–35	59	48,517	33	1387	92	1013
36–47	53	50,697	30	1614	92	979
48–59	47	48,512	26	1487	90	1042
Birth order						
1	60	73,998	36	2423	93	1961
2_3	55	93,620	30	2843	91	1889
4–5	46	24,051	23	592	76	214
6+	38	8220	14	124	45	30
Sex of the child						
Male	55	1,03,622	32	3090	91	2177
Female	55	96,267	31	2892	91	1917
Child lives with whom						
Respondent	55	1,97,975	31	5929	91	4056
Lives elsewhere	36	1914	16	53	62	38
Mother’s education						
No education	45	63,097	21	1512	81	688
Primary or less	54	29,394	29	993	89	509
Secondary or less	61	89,255	35	3014	93	2258
Higher Education	64	18,143	41	463	96	639
Caste						
SC^a^	57	37,351	30	659	91	1787
ST^b^	51	40,044	31	1503	NA	NA
Others	55	1,14,708	31	3723	91	2298
Religion						
Hindu	56	1,43,834	31	5316	88	1420
Muslim	50	31,616	31	591	71	115
Christian	56	16,439	58	65	87	35
Others*	73	8000	NA	NA	94	2524
Wealth quintiles^c^						
Poorest	47	52,266	17	878	72	44
Poorer	54	46,882	25	1337	83	185
Middle	58	39,965	29	1441	86	582
Richer	60	33,529	37	1231	87	985
Richest	64	27,247	40	1095	95	2298
Place of Residence						
Urban	58	48,257	34	2003	89	1477
Rural	54	151,632	29	3979	93	2617
Place of Vaccination						
Public	60	1,68,491	35	4683	92	3608
Private	56	14,434	37	623	93	422
Year of birth						
2010	44	15,030	26	1008	90	775
2011	48	40,212	28	1598	90	1014
2012	55	50,909	30	1492	93	1029
2013	61	49,569	39	1396	92	993
2014	63	34,656	35	488	87	281
2015	58	9446				
Region						
North	58	37,737				
Central	47	56,634				
East	63	41,587				
North East	50	29,179				
West	46	14,328				
South	61	20,424				

NA denotes not enough sample (less than 30); ^a^Scheduled caste; ^b^Scheduled tribe; ^c^wealth quintiles denote five different economic classes of India

^d^State with lowest hep-B vaccination coverage; ^e^State with highest hep-B vaccination coverage;

* Denotes the other religious groups like Sikh, Buddhist/Neo-Buddhist, Jain, Jewish, Parsi/Zoroastrian & No religion

Sex of the child did not show any substantial difference in the HBV vaccination whereas birth order of the child showed a differential. Children of first birth order showed the highest coverage of the vaccine than the older birth orders. Results suggested that with the increasing age of mothers, a drop had been observed in HBV vaccine coverage in India as well as in Gujarat but not in Punjab. The coverage of HBV vaccine was found to be higher among the children who lived with their mother than otherwise in India. Similarly, mother’s education showed considerable difference in the coverage of hepatitis B vaccine among children and the coverage was comparatively more among the children whose mothers were higher educated than their counterpart. Similarly, the education gradient held true for the two states as well. In Gujarat the HBV vaccination was notably low with only 21% coverage among the children of no educated mothers.

Table 4 gives the conditional probabilities of HBV vaccination among the children by background characteristics. The conditional probability that a child in urban area will receive HBV vaccine is 0.54 while that in the rural area is 0.49. The sex of the children shows the same chances of being HBV vaccinated. Wealth status showed a clear increasing probability over the gradients and the chance was highest (0.61) of being HBV vaccinated if the child belonged to the highest wealth quintile whereas the chance was lowest (0.42) among the poorest children. Of the two caste groups, children belonging to SC showed the higher chance (0.52) of being vaccinated than the ST children (0.45). The chance of vaccination was maximum (0.55) for the first birth order and eventually the chance was as low as 0.35 for the six and higher ordered births. The probability was observed maximum among the children aged 12–23 months followed by the older ages with the lowest chance of being vaccinated among the 48–59 months age group of children. It was found that the chance was 50% if the child lived with the respondent otherwise the chance was around 36%. Educational status of the mother also showed an increased chance of HBV vaccination among those children of higher educated mothers. Among all the children, the chance was lowest (0.41) among those whose mothers were not having any formal education.

**Table 4:** The conditional probability of receiving the three doses of hepatitis B vaccine among the children by background characteristics, India, 2015–16

Background Characteristics	Conditional probability
	Pr[Hep B=Yes|X]
Child’s age in months	
12–23	0.62
24–35	0.59
36–47	0.53
48–59	0.47
Birth order	
1	0.55
2_3	0.5
4_5	0.42
6+	0.35
Sex	
Male	0.5
Female	0.5
Child lives with whom	
Mother	0.5
Lives Elsewhere	0.36
Mother’s education	
No Education	0.41
Primary or less	0.48
Secondary or less	0.55
Higher	0.6
Caste	
SC	0.52
ST	0.45
Others	0.52
Religion	
Hindu	0.51
Muslim	0.46
Christian	0.44
Others*	0.63
Wealth quintiles	
Poorest	0.42
Poorer	0.48
Middle	0.53
Richer	0.56
Richest	0.61
Place of Residence	
Rural	0.49
Urban	0.54
Place of Vaccination	
Public	0.93
Private	0.07
Year of birth	
2010	0.06
2011	0.17
2012	0.25
2013	0.27
2014	0.2
2015	0.05
Region	
North	0.56
Central	0.44
East	0.57
North East	0.44
West	0.45
South	0.59

* Denotes the other religious groups like Sikh, Buddhist/Neo-Buddhist, Jain, Jewish, Parsi/Zoroastrian & No religion

Table 5 shows the estimated adjusted odds ratio values (AOR) from the logistic regression showing the empirical association between HBV coverage among the study children and its predictors. Age pattern of HBV vaccination showed that compared to the 12–23 aged children, children of older ages were less likely to receive the vaccine. As the data are a cross sectional data and the current age of the children varied between 12 to 59 months it could be said that children born earlier were less likely to receive the vaccine than those children who born later. This result is consistent with the bivariate result and reflects the recent awareness about the importance of HBV vaccination. Furthermore, it could be an indication of the increasing financial burden associated with the subsequent children over the period of time.

**Table 5:** Logistic regression estimates for hepatitis B vaccination, India, 2015–16

Predictors	Adjusted Odds Ratio	Significance Level	95% Confidence Interval		Marginal probability
Child’s age in months					
12–23^a^					0.61
24–35	0.95	0.071	0.90	1.00	0.60
36–47	0.94	0.093	0.88	1.01	0.59
48–59	0.96	0.380	0.88	1.05	0.60
Birth Order					
1^a^					0.62
2–3	0.91	0.000	0.88	0.93	0.59
4–5	0.82	0.000	0.78	0.86	0.57
6+	0.76	0.000	0.71	0.83	0.56
Sex of the child					
Male^a^					0.60
Female	1.02	0.147	0.99	1.05	0.60
Child lives with whom					
Mother^a^					0.60
lives elsewhere	0.41	0.000	0.27	0.62	0.39
Mother’s education					
No education^a^					0.54
Primary or less	1.23	0.000	1.17	1.29	0.59
Secondary or less	1.47	0.000	1.41	1.53	0.63
Higher	1.48	0.000	1.37	1.59	0.63
Caste					
SC^a^					0.61
ST	0.96	0.208	0.90	1.02	0.60
Others	0.93	0.001	0.89	0.97	0.59
Religion					
Hindu^a^					0.60
Muslim	0.83	0.000	0.78	0.89	0.56
Christian	0.90	0.130	0.79	1.03	0.58
Others	2.09	0.000	1.82	2.41	0.75
Wealth Quintiles					
Poor^a^					0.53
Poorer	1.26	0.000	1.21	1.32	0.59
Middle	1.41	0.000	1.34	1.49	0.61
Richer	1.50	0.000	1.40	1.60	0.62
Richest	1.88	0.000	1.73	2.03	0.67
Place of Residence					
Urban^a^					0.59
Rural	1.06	0.039	1.00	1.12	0.60
Place of Vaccination					
Public^a^					0.61
Private	0.75	0.000	0.71	0.81	0.54
Year of Birth					
2010^a^					0.48
2011	1.24	0.000	1.16	1.32	0.53
2012	1.67	0.000	1.55	1.80	0.60
2013	2.03	0.000	1.85	2.23	0.65
2014	2.22	0.000	2.00	2.47	0.67
2015	1.81	0.000	1.59	2.07	0.62
Regions					
North^a^					0.57
Central	0.88	0.000	0.83	0.94	0.54
East	1.89	0.000	1.77	2.02	0.71
North East	1.03	0.480	0.95	1.12	0.58
West	0.69	0.000	0.63	0.76	0.49
South	1.23	0.000	1.14	1.32	0.62

^a^denotes the reference category

Birth order of the children showed a statistically significant association with the vaccination. And higher ordered births showed a lower likelihood of HBV vaccination compared to the first. Sex of the child did not show a statistically significant association with HBV vaccination status. We observed mother’s educational status to be highly significant for HBV vaccination. Results suggested that children of not educated mothers were less likely to be HBV vaccinated than their counter part. Religion of the children and HBV vaccination showed a statistically significant association and Christian children were more likely to receive HBV vaccine than the Hindu children whereas the Muslim children were less likely to receive this particular vaccine. Children’s household wealth status had shown a strong association with HBV vaccination and the estimated AOR values suggested that those children from the richest households were 88% more likely than the poorest children and even the children from the middle class households were 41% more likely than the poorest children to receive the HBV vaccine. Region wise, the likelihoods of HBV vaccination were also different. It was evident that compared to the children from North India, children from West and Central part of India were less likely to receive the vaccine whereas the children from Eastern India were 89% more likely and children from South were 37% more likely to be immunized against hepatitis B. In contrast, children from the Central region were 12% less likely to receive this vaccine.

## Discussion

The study findings suggest that there is a huge disparity lies in the coverage of hepatitis B vaccine across the districts of India. The national and state average conceals large variation in the coverage of hepatitis B. Of the total 640 districts across India, 110 districts show a coverage rate of less than 40% only. A total of 11 districts, mostly from the north and north western parts of India could vaccinate less than 20% of the children with hepatitis B. Only 17 districts in India covered more than 90% of the total children with the three doses of hepatitis B. And in some districts, the coverage is as low as 5%. Of all the 640 districts, only two districts from Punjab and a district from Kerala showed a coverage rate of 100% of hepatitis B vaccination. This indicates that district level variation is enormous for this vaccination coverage. Similarly, state level variation is also very distinct across India. And among all the other doses of full immunization (DPT, Measles, BCG and Polio), the coverage of three doses of hepatitis B vaccine is lowest among the children.

This study finds a linkage between maternal health care utilization and an increased chance of hepatitis B vaccination across the districts. Previously, it has been studied that during antenatal care service and institutional delivery mothers are promoted to access the next level of health care services like immunization specially [25, 27]. From this study it is also evident that districts where more mothers utilized maternal health care services (PNC and ANC) show higher coverage of hepatitis B vaccination among the children. The spatial modeling also identified that districts where poor mothers received financial assistance under the JSY scheme is substantially associated with better immunization coverage in those districts. The district level exploration of the data identified those geographical pockets where hepatitis B vaccination among children is substantially low and districts with low coverage of hepatitis B vaccine are clustered regionally. Another interesting finding from the study tells that mother’s education is also associated with the increased chance of hepatitis B vaccination among the children. This suggests that educated mothers are more aware of their child’s health and health care utilization. And this could be the pathway to educate the mothers in terms of their child health across different sub population and simultaneously reduce the knowledge gap about hepatitis B vaccination along with other compulsory doses of immunization. The study findings also demonstrated the role of other contextual correlates which significantly determine the immunization status of the children in India.

In India, the coverage of hepatitis B vaccination is substantially low for a long time and still almost half of the children do not receive the doses of hepatitis B. Previous studies already argued about the importance of integrating hepatitis B vaccine along with the other compulsory doses of UIP [2, 10]. Some other studies found that there is inequity among the children in India in receiving the non-UIP vaccines from the private health care facilities and paediatricians often don’t prescribe the non-UIP vaccine doses because there are patients who cannot afford the doses bearing the market price [28]. On the other hand, health care access and economic status of the household largely determines access to UIP vaccine doses [29]. Similarly, physical distance to health facility and cost of the medical services do play a crucial role in patient’s care-seeking behaviour [30]. So governance should meet the inequity in the vaccination access among the poor and SC-ST population and in the geographically remote areas. In this direction, a number of studies performed the economic analysis and suggested incorporating hepatitis B vaccine in the national immunization program in India [31, 32].

Recently, India showed an improvement in terms of hepatitis B birth dose coverage but still the birth dose coverage is 45% only despite the high rate of institutional deliveries [11]. This is possibly because hepatitis B as the birth dose was not included for all institutional deliveries after the vaccine introduction [10]. One reason for low uptake of hepatitis B birth dose attributed to the high vaccine wastage as health staffs were often refrained from not opening a new 10 dose vial of the vaccine for a low number of deliveries that take place in the health facilities [10]. Studies from other country settings also suggest that weakness in policy development and implementation, poor communication and lack of effective training among health staffs to be the major reasons for the low coverage of the hepatitis B birth dose [33, 34]. A previous study for Vietnam finds the link to increased chance of receiving hepatitis B birth dose with community based pregnancy tracking, the perception regarding the immunization and perceived contraindications [35]. To scale up the rate of hepatitis B vaccination, the Open Vial Policy for birth dose of hepatitis B vaccine has been adopted as a part of the universal immunization program (UIP) and efforts are made to sensitize the health care staffs about hepatitis B birth dose administration.

The study has several limitations, largely due to lack of data. First, the study could not explore the cost associated with the hepatitis B vaccination among the children who received this vaccine as non-UIP vaccines from the market or private health care facilities. NFHS-4 lacked on the cost information. Second, the study did not cover the reason for not vaccinating the child against hepatitis B. Third, as a part of the survey, the interviewer collected the specific information on immunization of all the children 5 years prior to the survey from their mothers or care givers which are not as reliable as that of vaccination card due to recall bias. Fourth, due to lack of information, this study could not explore the factors which include access and availability of services for the hepatitis B vaccination coverage. Logistics, access to health care and trained human resources could potentially predict rate of hepatitis B vaccination across the districts like the other doses of UIP [36]. Despite the above data limitations, we conducted this study to examine the district level spatial heterogeneity of hepatitis B vaccination coverage and its micro level socio-economic and demographic predictors to inform policy and program actions at sub national level. Future studies should focus on perception regarding the immunization in the population implementing a qualitative study. Additionally, efforts should be made at the national and at sub-national level to understand the seroprevalence to determine the actual immune status among the children and provide an estimate of the prevalence helping the governance for proper interventions.

## Conclusion

This study contributes to the understanding of hepatitis vaccine coverage at the district level and examined the spatial clustering. Simultaneously, this study estimated the vaccine (hepatitis B) coverage across the sub population and examined the factors associated with the low coverage of hepatitis B vaccine. Improving maternal education and awareness about hepatitis vaccination and its schedule among the mothers can play a crucial role to help increase the coverage of immunization. As utilization of maternal health care utilizations (ANC, PNC and institutional delivery) among mothers showed a strong association with hepatitis B vaccination across the districts, promoting mothers to avail the maternal health care utilization during pregnancy can also play a pivotal role in those districts where maternal health care utilization is low and neglected. At the same time, health care staffs from the health centres should be trained and sensitized to promote the mothers about the uptake of this particular vaccine among their children and should be taught on the advantages of vaccinating their children and its due process.

As India is a geographically diverse country and 70% of the total population being rural, needs more careful monitoring and evaluation on the uptake of hepatitis B vaccine. This study identified the geographical cold spots where the vaccination coverage is substantially low and should be targeted to improve the vaccination coverage in those districts. As the maternal health care utilizations showed a significant association with the coverage of hepatitis B vaccine across the districts, Mothers from the poor wealth quintiles who are promoted to utilize institutional delivery through incentives under the JSY scheme should also be incentivised for their children’s vaccination. This incentivization should also follow the mothers across different socially excluded groups and under privileged sections of the society. Additionally, the “Janani Suraksha Yojana” platform could be used to increase the coverage of hepatitis B birth dose promoting the mothers from high risk population groups like the tribal communities and population groups residing in remote areas. Furthermore, they should be targeted and closely monitored for the uptake of hepatitis B vaccine along with other routine immunization doses [2, 16, 37].

## Data Availability

Data is freely available on the Demographic and Health Survey (DHS) website https://dhsprogram.com/data/dataset/India_Standard-DHS_2015.cfm?flag=1 and therefore, does not require any separate ethical approval.

## Appendix

**Table 6:** Children aged 12-59 months received the doses of hepatitis B vaccine, India, 2015-16

	Frequency	Percent
Received Hepatitis-b 1		
No	42,626	20.13
Reported on vaccination card	86,021	40.62
Reported by mother	67,458	31.85
Don’t know	3,784	1.79
Not Applicable	11,884	5.61
Received Hepatitis-b 2		
No	52,922	24.99
Reported on vaccination card	84,163	39.74
Reported by mother	59,020	27.87
Don’t know	3,784	1.79
Not Applicable	11,884	5.61
Received Hepatitis-b 3		
No	85,838	40.53
Reported on vaccination card	80,782	38.15
Reported by mother	29,485	13.92
Don’t know	3,784	1.79
Not Applicable	11,884	5.61
Total	2,11,773	100

**Table 7:** Moran’s *I* statistics of Hepatitis B vaccine coverage and socio-economic indicators in districts of India, 2015-16

Indicators	Moran’s *I* -value
Hepatitis B vaccine (%)	0.63(0.001)
Women with 10+ years of schooling (%)	0.67(0.001)
Full ANC (%)	0.68(0.001)
PNC (%)	0.56(0.001)
Mothers who received financial assistance under JSY(%)	0.72(0.001)
Institutional Births (%)	0.62(0.001)
Electricity (%)	0.63(0.001)
Drinking water (%)	0.46(0.001)
Births Registered (%)	0.65(0.001)
Breastfed child received adequate diet (%)	0.49(0.001)

## Abbreviations

AIC: Akaike Information Criterion
AIDS: Acquired Immuno Deficiency Syndrome
ANC: Antenatal Care
ANM: Auxiliary Nurse Midwife
AOR: Adjusted Odds Ratio
BCG: Bacillus Calmette-Guerin
DHS: Demographic Health Surveys
DPT: Diphtheria, Pertussis, Tetanus
EPI: Expanded Program on Immunization
HBsAg: Hepatitis B Surface Antigen
HBV: Hepatitis B Virus
HIV: Human Immunodeficiency Virus
JSY: Janani Suraksha Yojana
LHV: Lady Health Visitor
LISA: Local Indicator of Spatial Autocorrelation
NFHS: National Family Health Survey
OBC: Other Backward Class
OLS: Ordinary Least Square
OPV: Oral Polio Virus
PNC: Post Natal Care
PPS: Probability Proportional to Size
PSU: Primary sampling unit
SAR: Spatial Autoregressive Models
SC: Scheduled Caste
SEM: Spatial Error Model
SLM: Spatial Lag Model
ST: Scheduled Tribe
UIP: Universal Immunization Program
VPDs: Vaccine Preventable Diseases
WHO: World Health Organization

## Footnotes

1 The prevalence of hepatitis B surface antigen (HBsAg) tells about the positive surface antigen of the hepatitis B virus indicating the chronic carriers of hepatitis B virus.

2 According to the definition of NFHS-4, improved sources of drinking water include piped water, public taps, standpipes, tube wells, boreholes, protected dug wells and springs, rainwater and community reverse osmosis (RO) plants.

3 Breastfed children who received four or more food groups with a minimum meal frequency of 2-3 times a day, i.e., the children received solid or semi solid foods at least two- three times from the following food groups - a. infant formula, milk other than breast milk, cheese or yogurt or other milk products; b. foods made from grains or roots, including porridge or gruel, fortified baby food; c. vitamin A-rich fruits and vegetables; d. other fruits and vegetables; e. eggs; f. meat, poultry, fish, shellfish, or organ meats; g. beans, peas, lentils, or nuts; h. foods made with oil, fat, ghee, or butter
